# Massive sequencing of *Ulmus minor*’s transcriptome provides new molecular tools for a genus under the constant threat of Dutch elm disease

**DOI:** 10.3389/fpls.2015.00541

**Published:** 2015-07-20

**Authors:** Pedro Perdiguero, Martin Venturas, María Teresa Cervera, Luis Gil, Carmen Collada

**Affiliations:** ^1^Grupo de Investigación en Genética, Fisiología e Historia Forestal, Departamento de Sistemas y Recursos Naturales, Universidad Politécnica de MadridMadrid, Spain; ^2^Unidad Mixta de Genómica y Ecofisiología Forestal, Instituto Nacional de Investigación y Tecnología Agraria y Alimentaria/Universidad Politécnica de MadridMadrid, Spain; ^3^Departamento de Ecología y Genética, Centro de Investigación Forestal, Instituto Nacional de Investigación y Tecnología Agraria y AlimentariaMadrid, Spain

**Keywords:** Dutch elm disease, next-generation sequencing, SNPs, transcriptome, *Ulmus*

## Abstract

Elms, especially *Ulmus minor* and *U. americana*, are carrying out a hard battle against Dutch elm disease (DED). This vascular wilt disease, caused by *Ophiostoma ulmi* and *O. novo-ulmi*, appeared in the twentieth century and killed millions of elms across North America and Europe. Elm breeding and conservation programmes have identified a reduced number of DED tolerant genotypes. In this study, three *U. minor* genotypes with contrasted levels of tolerance to DED were exposed to several biotic and abiotic stresses in order to (i) obtain a *de novo* assembled transcriptome of *U. minor* using 454 pyrosequencing, (ii) perform a functional annotation of the assembled transcriptome, (iii) identify genes potentially involved in the molecular response to environmental stress, and (iv) develop gene-based markers to support breeding programmes. A total of 58,429 putative unigenes were identified after assembly and filtering of the transcriptome. 32,152 of these unigenes showed homology with proteins identified in the genome from the most common plant model species. Well-known family proteins and transcription factors involved in abiotic, biotic or both stresses were identified after functional annotation. A total of 30,693 polymorphisms were identified in 7,125 isotigs, a large number of them corresponding to single nucleotide polymorphisms (SNPs; 27,359). In a subset randomly selected for validation, 87% of the SNPs were confirmed. The material generated may be valuable for future *Ulmus* gene expression, population genomics and association genetics studies, especially taking into account the scarce molecular information available for this genus and the great impact that DED has on elm populations.

## Introduction

Owing to their long life cycles, forest tree species need to develop evolutionary mechanisms and processes to cope with different abiotic and biotic stresses. Plant responses to simultaneous stresses are complex, and may result in antagonistic, synergistic, or additive interactions (Suzuki et al., 2014). The results will depend on the timing, nature, and severity of each stress (Prasch and Sonnewald, 2015). Current climate change is drastically affecting Mediterranean regions and promoting the occurrence of numerous situations of combined environmental stresses. For example, reduction of water availability in the Iberian Peninsula, often accompanied by pest outbreaks, is causing important damage to its forests (Sánchez-Salguero et al., 2012).

Field elm (*Ulmus minor* Mill.) is a riparian noble hardwood tree which is affected by a multiple stress scenario like the one described above. On the one hand, climate change models predict an increase in temperature, aridity and extreme events throughout its natural distribution, composed mainly by central and southern Europe. Although the species is well adapted to a wide range of pluviometric conditions, with annual precipitation ranging from 350 to 900 mm as well as a 3- to 4.5-month summer drought period (Venturas et al., 2013b), this species is expected to face more severe and restrictive environmental conditions. On the other hand, and more importantly, Dutch elm disease (DED) is an important threat to elm populations. DED is a wilt disease caused by two fungi, *Ophiostoma ulmi* (Buisman) Nannf. (moderately pathogenic) and *O. novo-ulmi* Brasier (highly pathogenic). Two successive pandemics of DED that occurred during the twentieth century caused the death of most adult trees in many locations of Europe and North America, including those used as ornamental trees in urban areas (Brasier, 2000). The combined effects of climate change and DED, which is really difficult to control due to its high impact and fast expansion, will jeopardize the survival of the remaining elm forests during the next decades.

Tolerant elm genotype selection and breeding has been the most successful strategy for elm recovery (Santini et al., 2004; Solla et al., 2005a). Several breeding programmes for *Ulmus* sp. affected by DED have been developed since the disease appeared (Heybroek, 1993; Smalley and Guries, 1993; Santini et al., 2004). Usually Asian elms that show low susceptibility to the disease (f.i. *U. pumila* L. or *U. wallichiana* Planch.) have been used as a source of resistance in these breeding programs. The inherent adaptive capacity of *U. minor* has been investigated by the Spanish Elm Breeding and Conservation Programme (Martín et al., 2012). This programme has identified several Iberian genotypes with a high degree of tolerance to DED when they were artificially inoculated with an aggressive strain of *O. novo-ulmi* (Solla et al., 2005a; Martín et al., 2015). Genetic heritability of resistance has been shown both by crossing native and Asian elms (Smalley and Guries, 2000; Santini et al., 2011) and by crossing susceptible and tolerant Iberian *U. minor* genotypes (Venturas et al., 2014).

In spite of these results, the molecular knowledge of *Ulmus* sp. is scarce. The major information available is an EST database with 52,823 unique transcripts including defense response genes activated in *U. minor* leaves after egg laying by the elm leaf beetle (*Xanthogaleruca luteola* Mull.) or the application of methyl jasmonates (Büchel et al., 2012). Only a few genes from *U. americana* L. upregulated during *O. novo-ulmi* infection have been identified and analyzed using *in vivo* or *in vitro* interaction (Nasmith et al., 2008; Aoun et al., 2010). Noteworthy, these studies did not use tolerant genotypes. Regarding molecular markers, a collection of nuclear microsatellites (Simple Sequence Repeats, SSRs) have been developed for *Ulmus* sp. (Whiteley et al., 2003; Collada et al., 2004; Zalapa et al., 2008) and have been commonly used in different diversity studies (Zalapa et al., 2009; Brunet et al., 2013; Venturas et al., 2013a; Fuentes-Utrilla et al., 2014a,b). In contrast, no single nucleotide polymorphisms (SNPs) have yet been described for these species.

Knowledge of a species transcriptome is very useful for researchers who work with non-model plant species (Garg and Jain, 2013). Next-generation sequencing (NGS) technologies have greatly facilitated transcriptome sequencing of species with scarce or without prior gene sequence information (Ekblom and Galindo, 2010; Neale and Kremer, 2011). These NGS techniques have been used by forest researchers for gene discovery and annotation, large-scale expression analysis and marker discovery (Novaes et al., 2008; Bräutigam and Gowik, 2010; Parchman et al., 2010; Ge et al., 2011; Pinosio et al., 2014). Taking into account that SNPs are the most abundant genetic variations in genomes, this information is highly useful in breeding and conservation programs. Thus, SNP-based markers are replacing or being combined with SSRs and other molecular markers for construction of dense genetic maps of trees and other plants (Pavy et al., 2008; Vezzulli et al., 2008; Chancerel et al., 2011; Gaur et al., 2012) allowing a more effective dissection of traits of interest.

The available *Ulmus* sp. molecular resources are clearly insufficient to preserve and manage the threatened elm populations (Bernier et al., 2015). Large-scale development of SNP markers would allow researchers to use more efficient and powerful tools in the management and conservation of *U. minor* genetic resources and those of other Ulmaceae species. In this study we use GS-FLX 454 pyrosequencing to study elm transcriptome responses to abiotic and biotic stresses. We designed this study to (i) construct a non-normalized library covering responses to the most important biotic and abiotic stresses suffered by three *U. minor* genotypes with different degrees of tolerance to DED, (ii) perform the assembly and functional annotation of the transcriptome, (iii) identify those genes potentially involved in abiotic and biotic stress responses, and (iv) identify potential functional gene-based markers (SNPs). The three genotypes were inoculated with *O. novo-ulmi* and *O. ulmi*, the causal agents of DED, as well as with the endophytic fungus *Daldinia concentrica* (Bolton) Cesati & de Notaris. These genotypes were also exposed to water stress. In order to obtain the molecular response of all plant organs, the different tissues (roots, stems, and leaves) were collected separately, and their RNA extracted. Afterward, RNA of all tissues was mixed in equal proportions and all samples pooled for sequencing.

## Materials and Methods

### Plant Material

Clonal material from three Iberian *U. minor* genotypes with different degrees of tolerance to DED was used for this study. Two of these genotypes, M-DV4/5 from Dehesa de la Villa (Madrid) and J-CA2 from Sierra de Cazorla (Jaén), showed a moderate to high degree of tolerance to DED, and the third, TO-AL1 from Algodor (Toledo), was highly susceptible to the disease. These genotypes have been widely used as parents in controlled crossed carried out within the Spanish Elm Breeding and Conservation Programme (Venturas et al., 2014). Plants were propagated by stem cuttings and grown in 2 L pots (1:3 perlite:peat substrate, v/v) at Puerta de Hierro nursery (Madrid). Plants were well watered. One-year-old plants were subjected to four different treatments: drought and three different inoculations with *O. novo-ulmi* (strain CU-HU), *O. ulmi* (PM-VA2) or *D. concentrica* (X23-1).

One plant per genotype and treatment was collected at every sampling point. For the water stress treatment, plants were watered to field capacity at the beginning of experiment and were collected after 48 h and 7 days without irrigation. For the analysis of elm response to *O. ulmi* and *O. novo-ulmi*, trees were inoculated with 10^6^ spores ml^-1^ according to Solla et al. (2005a) and plant material was collected 24 h and 7 days post-inoculation. These plants showed wilting symptoms after inoculation, especially the susceptible clone, but this process was not quantified. For the analysis of elm response to *D. concentrica*, inoculation was conducted removing a small piece of bark (1 cm^2^) from the main stem and inserting *D. concentrica* mycelium in the cambium. Immediately after inoculation the wound was sealed with parafilm. Plant material of this treatment was also collected 24 h and 7 days after inoculation. Unstressed plants of the three genotypes were harvested at the end of treatments. The plants were dissected into leaves, stems, and roots. Tissues were immediately frozen in liquid nitrogen and stored at -80°C.

### RNA Extraction and cDNA Synthesis

Total RNA for each sampling point was extracted separately from roots, stems, and leaves of each sampled plant following the CTAB–LiCl precipitation method (Chang et al., 1993). Equal amounts of total RNA from roots, stems, and leaves from all the plants were mixed to form one RNA pool to maximize the diversity of transcripts obtained. RNA was DNase treated to remove genomic contamination and purified using the Qiagen RNeasy kit (Qiagen, CA, USA). cDNA was synthesized from 5 μg de RNA using SMART approach (Zhu et al., 2001). Non-normalized cDNA was purified using QIAquick PCR Purification Kit (Qiagen, CA, USA).

### Roche 454 Sequencing and Assembly

Five μg of non-normalized cDNA were used for library construction and sequencing using GS-FLX Titanium System (Roche). The sample was nebulized, adaptor-ligated, and pyrosequenced (1 plate). Sequencing was performed by the Lifesequencing service (Valencia University, Spain). Sequences were *de novo* assembled with Newbler version 2.3 (Roche). Using this software reads were filtered by successive steps. In the first step low quality reads were rejected. In a second step the remaining reads were trimmed and classified. Those that were too short (reads lower than 100 pb) and outliers (problematic reads such as chimeras) were rejected, whereas singletons (no significant overlapping with other reads) were considered unique transcripts at this point. Finally, the remaining repeated, partially assembled and assembled reads were used to construct isotigs. The minimum match length for overlapping was fixed in 40 pb and the minimum identity percentage in 90% maintaining the rest of parameters as default.

### Sequence Analysis and Annotation

The transcriptome assembly (isotigs from assembled reads and singletons) was used as query for a BLASTx in two steps. Initially all sequences were compared with RefSeq proteins from highly annotated genus (*Arabidopsis, Populus, Oryza, Solanum, Glycine, Vitis, Medicago, Ricinus*, and *Malus*) using an *E*-value threshold of 10^-5^. Sequences without blast results were compared in a second step with the remaining plant RefSeq protein database. In order to estimate the number of different Unigenes identified, two independent steps of filtering were performed to remove any possible source of contamination from the final transcriptome and to avoid redundancy as far as possible. On the one hand, sequences without blast results were analyzed using SeqTrimNext software (advanced version of SeqTrim, Falgueras et al., 2007) with a specific template customized for Roche 454 GS-FLX plant transcriptomic data. To identify and reject sequences from inoculated organisms an additional comparison was performed with a database containing sequences from *O. novo-ulmi* and *O. ulmi* genomes (Forgetta et al., 2013; Khoshraftar et al., 2013), from *D. concentrica* (48 sequences in GenBank database) and sequences from *D. eschscholzii* genome (Ng et al., 2012). By this analysis a set of sequences that present homology with inoculated organisms were identified and analyzed with fungus RefSeq protein database, and also with protein dataset from the last annotation available for *O. novo-ulmi* (Comeau et al., 2015). Contaminant sequences from other common microorganisms and putative artifacts were identified and rejected. On the other hand, sequences sharing the best blast hit were considered the parts of same Unigene.

BLASTx annotations were conducted with version V.2.7.2 of Blas2GO for the Gene Ontology (GO) assignment. GO terms coming from the three different GO ontologies (biological process, molecular function and cellular component) were analyzed separately. Annotation results were filtered according to Plant GO-Slim, a reduced version of GO adapted for plants. The main goal of this analysis is to provide a more general and homogeneous perspective of the GO Terms found in a sample. Enzyme codes (ECs) and KEGG (Kyoto Encyclopedia of Genes and Genomes) pathways annotation were generated in Blast2GO from the direct mapping of the GO terms to their EC equivalents. Annotation inferred by homology with protein families, domains, and functional sites from secondary databases were obtained using InterProScan software implemented in Blast2GO.

### SNP Identification and Validation

To detect SNPs, we used the consensus assembly generated from all sequencing runs as a reference sequence to which individual reads were aligned using GS Mapper. GS Reference Mapper only scores polymorphisms where two or more reads contain the variant allele. Only SNPs detected differences that were classified as “High Confidence” were considered for the analysis. These differences are characterized by the following features: (1) there must be at least three non-duplicate reads with the difference; (2) there must be both forward and reverse reads showing the difference, unless there are at least five reads with quality scores over 20 (or 30 if the difference involves a 5-mer or higher).

In order to validate a sample of detected SNPs, we designed primers to amplify 13 isotigs randomly selected. Genomic DNA was used as template, this was isolated according to Doyle (1990) from leaves of three different genotypes included in library constructions. Specific primers for PCR amplifications were designed using Primer 3 software (Rozen and Skaletsky, 1999; Supplementary Table S1). Sequencing chromatograms were aligned and analyzed with BioEdit software (Hall, 1999). SNPs were identified as overlapping nucleotide peaks and validated.

### Data Accessibility

Files containing 454 reads are available from National Center for Biotecnology Information (NCBI) Short Read Archive [GenBank: accession number SRR1687227]. The assembled transcriptome, annotations, and SNP identification are available from Dryad entry doi: 10.5061/dryad.ps837.

## Results

### Sequencing and Assembly

**FIGURE 1:**
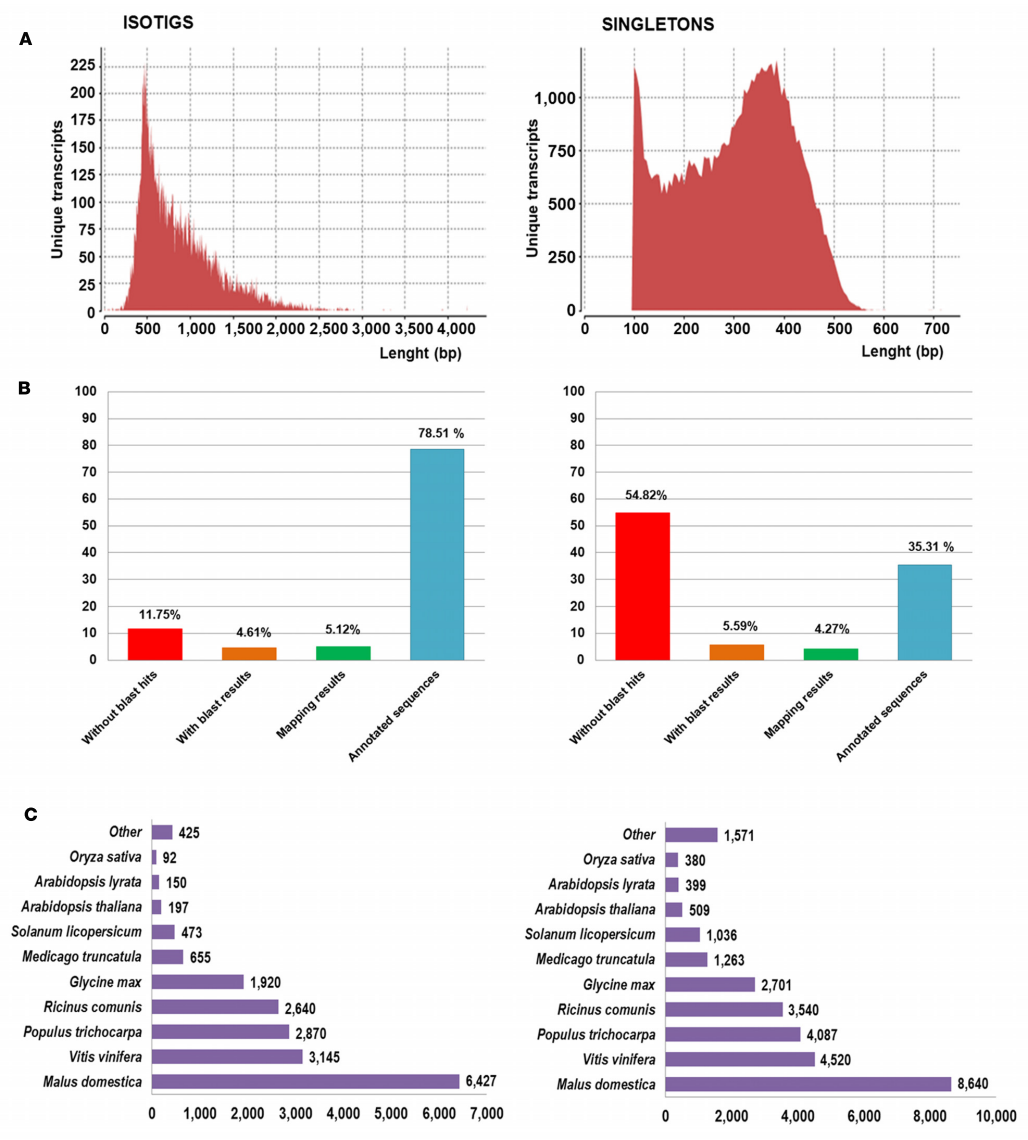
**Sequence length and blast results distribution for isotigs and singletons. (A)** Sequence length distributions for unique transcript identified after assembly **(B)** Proportion of *Ulmus minor* transcript without homology with plant proteins (without blast hits), with homology but no associated function (with blast results), with Gene Ontology (GO)-terms but not significantly associated (mapping results) and with GO-terms significantly associated (annotated sequences). **(C)** Best blast hit species distribution with number of transcript identified for each species.

The non-normalized library prepared using RNA from different genotypes, organs, and treatments included a total of 971,002 reads. During alignment, Newbler assembler trimmed the reads, and a total of 22,963 reads, corresponding to outlier or too short reads, were deleted. Before this filter, high quality reads were processed in two groups: (a) reads with significant overlapping between them (>40 bp and >90% identity) were *de novo* assembled resulting in 21,524 isotigs; the average length for all isotigs was 861 pb with a 30% of sequences ranging from 400 to 600 bp, and the N50 value for isotigs was 1,000 pb. (b) A set of 63,405 reads over 100 pb after being trimmed with Newbler did not show sufficient overlap with any other read and they were classified as singletons; the average length of these sequences was 303 pb (**Figure 1A**). Altogether, a total of 84,929 unique transcripts were obtained covering 37.77 Mbp.

### Annotation

*Ulmus minor* transcriptome (isotigs and singletons) was compared with GenBank RefSeq protein database. Blast results were found for a total of 47,640 unique transcripts. 88.14% of isotigs showed homology with proteins from Viridiplantae; for 18,707 sequences blast results were found within most common model species, whereas results were assigned for 287 isotigs in second round. Regarding singletons, blast results were obtained for 27,231 and 1,415 at the first and second round, respectively, reaching the 45.18% (**Figure 1B**). Putative isoforms or different regions from the same gene were grouped in unigenes according to blast results. The 47,640 unique transcripts with blast results identified a total of 32,152 different proteins as best blast hit which were considered different unigenes. Sequences with no blast hits were analyzed using SeqTrimNext. From 37,289 unique transcripts without homology, a total of 26,277 were accepted by SeqTrimNext and considered Unigenes without homology. On the contrary 11,012 unique transcripts (10,827 singletons and 185 isotigs) were identified as artifacts or contaminations and were rejected. Noteworthy, a little group of 181 unique transcripts within these sequences showed similarities with regions from genome of inoculated organisms (Supplementary Table S2). Specifically, 146 unique transcripts found a best blast hit with *O. ulmi* genome, 6 showed the highest homology with *O. novo-ulmi*, and the remaining 29 sequences aligned best with *D. eschscholzii* genome. Commonly its homology resulted in a partial alignment with a low percentage of coverage, lower than 25% in 86 sequences. Fifty-six sequences out of 181 (31%) showed homology with proteins from fungi, out of which 20 unique transcripts showed homology with *O. novo-ulmi* proteins from Comeau et al. (2015). In summary, regarding *U. minor* transcriptome, a total of 58,429 unigenes were identified. This data represents 1.3 to 1.7% of the *U. minor* genome, considering a genome size of 2,078.25 Mbp/1C (Loureiro et al., 2007). **Figure 1C** shows the species with high number of best blast results.

**FIGURE 2:**
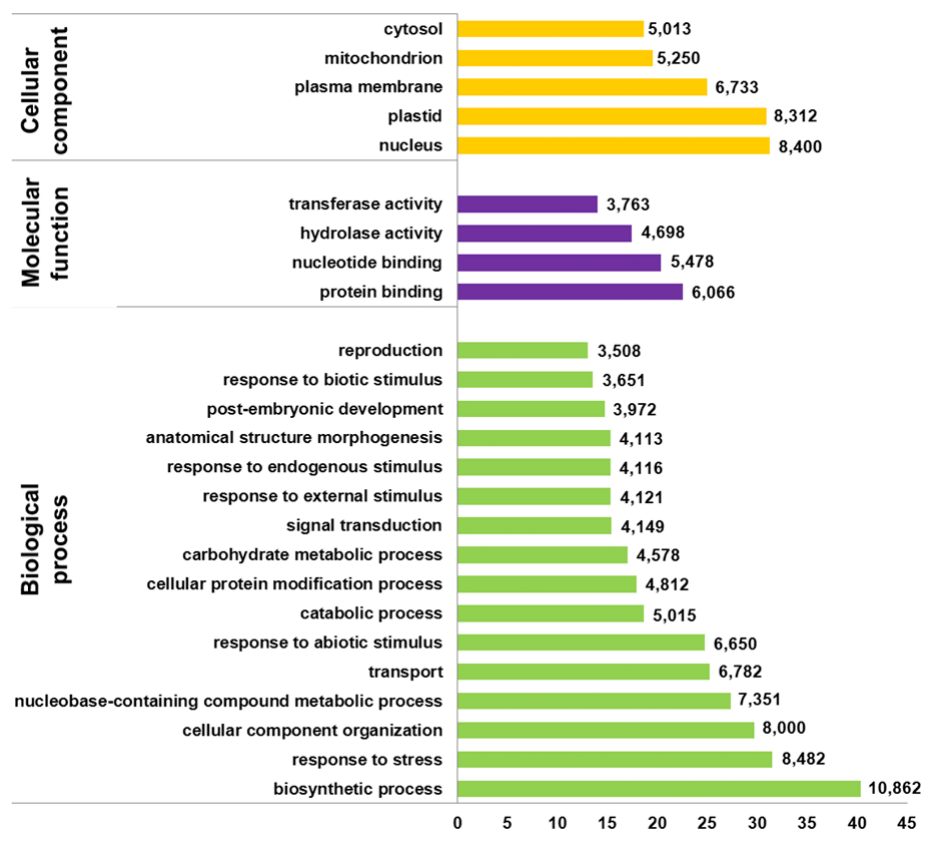
**Distribution of most abundant GO terms found in filtered version of *U. minor* transcriptome.** The proportion and number of unigenes in the three GO categories “biological process,” “molecular function,” and “cellular component” is reported for categories containing at least 3,000 unigenes.

**FIGURE 3:**
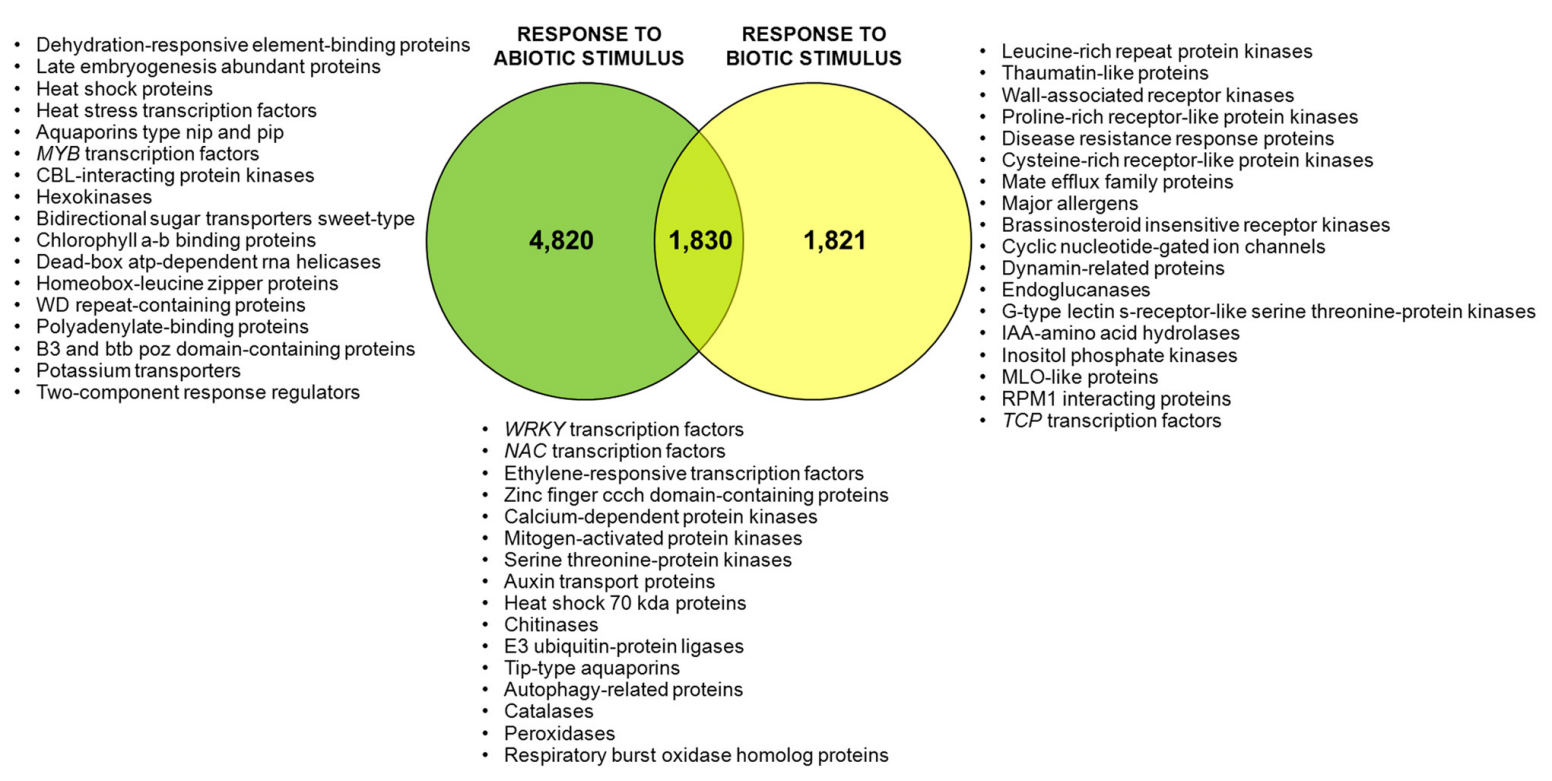
**Venn diagram for unigenes related with abiotic or biotic stress stimulus.** Number of unigenes annotated with each Go-term “response to abiotic stimulus” and “response to biotic stimulus” and with both GO-terms. Proteins identified exclusively for each group are shown.

Using Blast2GO gene ontology terms were assigned to unique transcripts for which a BLASTx match was obtained. A total of 26,903 unigenes were annotated with 266,843 GO terms. Most of the assignments (153,693; 58%) belonged to the biological process category, while cellular component category reached a 25% (67,419 GO terms) and molecular function was represented at a lower extent with 45,730 GO terms (17%). The distribution of most abundant GO annotations in the different functional categories evidenced a representative transcriptome of *U. minor* (**Figure 2**). All terms related to responses to stimulus are among the most abundant GO terms. Six-thousand six-hundred and fifty unigenes were associated to “response to abiotic stimulus” (GO: 0009628) whereas 3,651 unigenes were annotated for “response to biotic stimulus” (GO: 0009607). In both groups specific unigenes commonly described in response to different stresses were found; for instance, numerous *MYB* transcription factors, dehydration-responsive element-binding proteins, heat stress transcription factors, or late embryogenesis abundant proteins were identified specifically in “response to abiotic stress” group whereas leucine-rich repeat protein kinases, thaumatin-like proteins, disease resistance response proteins or wall-associated receptor kinases were commonly found in “response to biotic stress” group. One-thousand eight-hundred and thirty of these unigenes shared both GO terms which include numerous transcription factors families such as *NAC, ERF*, or *WRKY* as well as genes involved in common signal transduction pathways as calcium-dependent or serine threonine-protein kinases (**Figure 3**). Twenty-one isotigs were constructed with more than 1,000 reads. Among them 12 were associated with response to abiotic or biotic stimulus (**Table 1**).

**Table 1:** Sequences with more than 1,000 reads after assembly.

Sequence	N° reads	Description	Protein	Species	*E*-value	Abiotic or biotic
isotig08529	19,048	Senescence-associated protein	XP_003614399	*Medicago truncatula*	2.77E-20	
isotig13329	15,020	Metallothionein-like protein type 2	XP_002299873	*Populus trichocarpa*	1.47E-10	
isotig07130	2,395	Pectin methylesterase 5 family protein	XP_002304257	*P. trichocarpa*	2.71E-156	Biotic
isotig13408	2,265	No hits found	–	–		
isotig21287	1,951	Hypothetical protein	XP_002865071	*Arabidopsis lyrata*	8.24E-07	Abiotic
isotig13084	1,832	Kda class i heat shock protein	XP_002298362	*P. trichocarpa*	6.40E-20	Abiotic
isotig13342	1,766	No hits found_Rejected SeqTrim	–			
isotig02731	1,686	Chlorophyll a–b binding protein	XP_004248217	*Solanum lycopersicum*	2.03E-139	Abiotic
isotig21296	1,605	60s ribosomal protein l29-1	XP_002519200	*Ricinus communis*	4.85E-32	
contig07984	1,478	Mitochondrial protein, putative	XP_003588355	*M. truncatula*	1.37E-76	
contig06910	1,475	No hits found	–	–		
contig23572	1,426	Phosphoprotein ecpp44-like	XP_004238411	*S. lycopersicum*	4.71E-08	Abiotic
isotig07047	1,402	Bifunctional epoxide hydrolase 2-like	XP_003550367	*Glycine max*	1.34E-44	
contig22192	1,301	Catalase isozyme 1	NP_001268098	*Vitis vinifera*	0	Abiotic and biotic
isotig03470	1,270	Galactinol synthase family protein	XP_002265947	*V. vinifera*	0	Abiotic
isotig20888	1,156	Kda class i heat shock protein	XP_002530396	*R. communis*	2.69E-61	Abiotic
isotig13264	1,149	Plastocyanin family protein	XP_002510603	*R. communis*	5.77E-74	Abiotic
contig09765	1,120	Kda class i heat shock protein	XP_003618790	*M. truncatula*	1.74E-64	Abiotic
isotig18771	1,046	Calmodulin-like	XP_006378996	*P. trichocarpa*	2.84E-100	Abiotic
isotig02650	1,014	Thioredoxin h2-like	XP_008361428	*Malus domestica*	1.66E-58	Abiotic and biotic
contig07917	1,005	40s ribosomal protein s4	XP_002515668	*R. communis*	8.65E-166	

*For each sequence the information shown is: the number of reads, protein description, best blast hit including accession number of protein from RefSeq database, species and *E*-value, and information relative to annotation when sequences were associated to response to abiotic or biotic stimulus.*

Enzyme codes and KEGGs were also assigned to unigenes using Blast2GO. Nine-hundred and seven different ECs were identified and were included in 146 KEGGs pathways. Transferases and oxidoreductases were the most represented enzymes (33 and 27%, respectively), followed by hydrolases (17%). “Purine metabolism” with 55 ECs and “starch and sucrose metabolism” with 41 different ECs were the most represented pathways. Some pathways identified are relevant according to their implications in response to pathogens; the most significant pathways were “Phenylalanine metabolism” (represented by 25 ECs), alpha-linolenic acid metabolism (10 ECs) which results in jasmonate biosynthesis, cutin, suberine, and wax (4 ECs), flavonoids biosynthesis pathways (15 ECs), and 2 ECs (EC:2.7.10.2; protein-tyrosine kinase and EC:3.1.3.16 phosphatase) assigned to several genes involved in T-cell receptor signaling pathway.

### SNP Identification

Taking into account the use of three different genotypes for transcriptome construction a calling focused in identification of genetic variation was performed. GS Reference Mapper (454 Life Science) software was used to identify polymorphisms among ESTs by aligning individual reads against contigs from the assembly. Considering only high confidence variation, 7,125 different isotigs show a total of 30,693 polymorphic positions. Among these polymorphic variations 27,359 correspond to SNPs, 2,970 were classified as indels, and other type of variations were identified in the remaining 364 polymorphic sites. Transitions were the most common variation among SNPs, founding similar percentage regarding the type of substitution. The number of transversions identified was lower although also important (**Table 2**). To validate SNPs a sample of 13 isotigs randomly selected were PCR amplified from genomic DNA from the three genotypes used in transcriptome analysis. Each amplicon was sequenced bidirectionally (forward and reverse) using standard dideoxy-based sequencing. Long introns were observed in some genes, which were excluded from analysis during validation. From 142 identified SNPs, 123 were confirmed (87%), whereas 19 were non-validated. Also, an important number of not expected SNPs were observed during validation step (14 SNPs; **Table 3**, Supplementary Table S3).

**Table 2:** Changes observed in high confidence single nucleotide polymorphisms (SNPs).

Mutation types	Change	N°	Total N°
Transitions			16,579
	a–g	4,080	
	g–a	4,007	
	t–c	4,189	
	c–t	4,303	
Transversions			10,765
	a–c	1,194	
	a–t	1,665	
	c–a	1,344	
	c–g	1,170	
	g–c	1,120	
	g–t	1,481	
	t–a	1,552	
	t–g	1,239	
Other	^∗^ – N or N – ^∗^	15	15

*Number of detected changes for each variation type. N corresponds to SNPs for which three different nucleotides were observed.*

**Table 3:** Validation of SNPs with conventional sequencing.

Amplicon	Sequence	Description	Number of predicted SNPs	Validated	Non-validated	New SNPs	Abiotic or biotic stress
Amplicon1	contig02491	Flavonoid 3 -monooxygenase-like	11	11	0	4	Abiotic
Amplicon2	contig25532	Protein chloroplastic-like	10	10	0	0	
Amplicon3	isotig02731	Chlorophyll a–b binding protein chloroplastic-like	14	13	1	0	Abiotic
Amplicon4	isotig11158	Snf7 family protein	10	7	3	2	
Amplicon5	isotig11256	Mannose-1-phosphate guanylyltransferase alpha-like	9	9	0	3	Abiotic and biotic
Amplicon6	isotig11583	Eukaryotic initiation factor 4a-11	9	3	6	1	Abiotic
Amplicon7	isotig13076	Lipid-transfer protein dir1	10	10	0	1	Abiotic
Amplicon8	isotig13078	Cationic amino acid transporter vacuolar-like	10	9	1	1	Biotic
Amplicon9	isotig13259	Transcription factor myb44-like	14	13	1	0	Abiotic and biotic
Amplicon10	isotig13272	Anthocyanidin reductase	13	12	1	0	
Amplicon11	isotig18769	Atp synthase subunit	10	10	0	2	Abiotic
Amplicon12	isotig19674	Probable aldo-keto reductase 1	10	10	0	0	
Amplicon13	isotig20847	—NA—	12	6	6	0	
**Total**			**142**	**123 (87%)**	**19 (13%)**	**14**	

*Number and percentage of validated, non-validated and new SNPs for each of the amplicons from the tree genotypes (J-CA2, M-DV4/5, and TO-AL1) sequenced with dideoxy-based methods. Sequences associated with response to abiotic or biotic stimulus are shown.*

## Discussion

The aim of this work was to obtain the first transcriptome from *U. minor* in response to abiotic and biotic stress responses emphasizing on DED. Elm genotypes with different degrees of tolerance to DED were subjected to a drought treatment or inoculated with *O. ulmi, O. novo-ulmi*, or *D. concentrica* in order to induce transcription in response to stress. Whole plants were used in the research taking into account that spores of DED fungi germinate within stem, where hyphae grow and spread through xylem vessels inducing their blockage and cavitation. This results in foliar wilting, and in many occasions, the subsequent tree death. Additionally, DED fungi are also capable of spreading from tree to tree via root anastomosis (Neely and Himelick, 1963; Ghelardini and Santini, 2009). Therefore, we were interested in studying the transcriptome response of roots, stems and leaves.

To date no full genome from an elm species has been sequenced, and the genetic basis for tolerance to DED of *Ulmus* is practically unknown. Most of the public available information was produced to analyse *U. minor* molecular response to *Xanthogaleruca luteola* using 454 pyrosequencing methodologies to sequence different ESTs libraries (Büchel et al., 2012). This beetle can cause important damage to elm trees by defoliating them; however, the consequences of this pest are less devastating than those produced by DED which is capable of killing full elm populations (Brasier, 2000). Several works have analyzed DED from a physiological, metabolic or anatomical perspective (e.g., Martín et al., 2005b, 2009; Solla et al., 2005b; Ďurkovič et al., 2013; Venturas et al., 2014), however few studies have investigated this disease from a molecular point of view. The published works focused on molecular response to DED used northern blots, SSH, or RT-PCR to identify and study the expression pattern of a reduced number of genes mainly during *in vitro* interaction between elms callus cells and *O. novo-ulmi* (Nasmith et al., 2008; Aoun et al., 2009, 2010).

### Sequencing and Assembly

The *U. minor* transcriptome of this study represents the largest molecular source of information from *Ulmus* genus obtained up to date. This work has significantly contributed to increasing the number of high quality reads using 454 pyrosequencing from 361,196 previously obtained by Büchel et al. (2012) to 971,002. After assembly we obtained 84,929 unique transcripts with 74.7% singletons which are consistent with previous works based on non-normalized library sequencing with GS-FLX (Wall et al., 2009). Once sequences from contaminant organisms and redundancy were removed, a total of 58,429 unigenes were identified. The high number of unigenes here obtained from plants submitted to stress increase the scarce information available about molecular response to DED. To date only 314 unisequences were isolated from *U. americana* calli inoculated with *O. novo-ulmi* (Aoun et al., 2010). It is to be noted that 88% of these unisequences have been identified in the *U. minor* transcriptome here generated. Therefore, this transcriptome represents an important contribution to advance molecular knowledge on *Ulmus* response to DED.

### Annotation

Blast results were associated only to 56% of unique transcripts which shows the current lack of information on *Ulmus* genome. Remarkable differences in blast results were observed between isotigs and singletons. A high percentage of blast hits were found in isotigs (88.3%) in contrast to the lower percentage of blast results obtained for singletons (45.2%). This difference is probably associated with the shorter length of singletons which has been negatively correlated with annotability of sequences (Götz et al., 2008); the average sequence length for isotigs is 821 pb whereas for singletons is 303 pb. In previous works using the same sequencing methodologies a high percentage of singletons mapped with the reference genome (Wall et al., 2009) or their presence in the original sample were validated by PCR (Meyer et al., 2009). Singletons were ignored in annotation step by Büchel et al. (2012) during the analysis of *U. minor* libraries. However, our blast results highlighted a high proportion (45%) of singletons with homology with proteins from plants, many of which were not present in isotigs. Thus, in our transcriptome singletons could represent unique genes expressed at low levels taking into account that a non-normalized library was used as template. The presence of artifacts was solved as much as possible including the SeqTrimNext step. By this software 11,012 unique transcripts without blast results (10,827 singletons and 185 isotigs) were rejected using different criterion including a database for specific contaminants. Very few of these unique transcripts showed homology with *O. novo-ulmi, O. ulmi*, or *Daldinia* sequences (181 unique transcripts), which reflects that the transcripts of these inoculated organisms were present in low percentage in the samples. The pooling of numerous samples and the inclusion of whole plants, not restricted to the inoculation zone, may explain this scarce presence of contaminant sequences. Additionally, the spread of the fungi could be limited by plant size as the study was performed on one-year-old ramets (Solla et al., 2005b).

The species with high proportion of best blast results were *Malus domestica* Borkh., *Vitis vinifera* L. and *Populus trichocarpa* Torr. & A. Gray, and *Morus notabilis* Scneid. The number of genes deduced from the genome of these species were 57,386, 33,514, 45,654, and 29,338, respectively (Tuskan et al., 2006; Jaillon et al., 2007; Velasco et al., 2010; He et al., 2013). A similar number of genes would be expected in *U. minor*, thus the number of unigenes identified in this transcriptome (58,429) may be overestimated, suggesting a certain degree of redundancy. Moreover, considering that several fungal endophytes naturally colonize elm trees (Martín et al., 2013), it could be possible that some unigenes without blast results correspond to genes from endophytes as their genetic information is scarce in databases.

Numerous genes were annotated with GO terms associated with environmental stress. More than 3,000 unigenes were associated with each GO term related to responses to stimulus, including abiotic and biotic ones. A set of 1,830 genes share both GO terms (abiotic and biotic stress) which could point at components involved in the crosstalk between both responses (Glombitza et al., 2004; Fujita et al., 2006). Several genes and transcription factors commonly described during molecular response to environmental stresses have been identified in the transcriptome. Expression analysis was not an initial objective of our work, therefore were used pooled samples. However, considering that the majority of samples added to the mix were inoculated, the number of reads could give an idea of transcript enrichment in stress responses. It is noteworthy that more than 50% of genes with more than 1,000 reads were annotated with GO terms “response to abiotic” or “response to biotic” stimulus. Also, 10 out of 11 isotigs identified into the transcriptome are the orthologs of *U. americana* ESTs for which a notable upregulation was observed by RT-PCR during *in vitro* interaction with *O. novo-ulmi* (Aoun et al., 2010).

Within ECs and KEGGs the pathway “Phenylalanine metabolism” with 25 different ECs was remarkably represented. A central role of this pathway in the resistance to the wilt fungus *Verticillium dahliae* has been described in cotton (Xu et al., 2011). The enzyme phenylalanine ammonia lyase (PAL; EC: 4.3.1.24) has been widely used in studies focused on DED at two levels, measurement of enzymatic activity (Corchete et al., 1993) and induced transcripts (Nasmith et al., 2008). Correlation between PAL gene expression and accumulation of lignin, suberine, and phenols was observed by Aoun et al. (2009). Into this transcriptome, four ECs were identified in biosynthesis pathway of cutin, suberine, and wax and 15 ECs in flavonoids biosynthesis. The final products from these pathways have been described as resistance factors (Duchesne et al., 1985; Sticklen et al., 1991; Martín et al., 2005a). Another important pathway identified related with defense was the alpha-linolenic acid metabolism pathway that results in jasmonate biosynthesis, a plant hormone with a central role during biotic stress responses (Santino et al., 2013). It is remarkable that within T-cell receptor signaling pathway only two ECs were identified (EC:2.7.10.2 protein-tyrosine kinase and EC:3.1.3.16 phosphatase) but the number of unigenes associated to these activities (201 and 81 respectively) was higher in proportion to other pathways.

### SNP Identification

The present work represents the first massive identification of SNPs and other polymorphism in *Ulmus* species. The use of three different genotypes allowed the identification of 30,693 high fidelity polymorphic positions in 7,125 different isotigs. A validation of SNPs in a subset of isotigs by conventional PCR confirmed 87%. Similar results both in identification and validation of SNPs were observed in *Eucalyptus grandis* (Novaes et al., 2008). The majority of these new SNPs were identified by GS Reference Mapper, but they do not follow the parameters to be considered high confidence variation. Extrapolating the results to the complete transcriptome we could expect approximately 22,500 valid SNPs, a high percentage of them localized in genes involved in abiotic or biotic response. Seven isotigs amplified for validation were associated with “response to abiotic” or “response to biotic” stimulus GO terms.

## Conclusion

The transcriptome collection represents the major genetic resource for *U. minor* focused on biotic and abiotic stress responses, including DED. This wilt disease strongly affects *Ulmus* species causing the death of numerous adult trees, which could cause genetic impoverishment of elm populations. Considering that the genotypes used as starting material showed different degree of tolerance to DED, a large number of genes and enzymes identified could be potentially involved in molecular pathways that confer this trait. In addition, the inclusion of a water stress treatment increased the number of genes involved in response to dehydration activated as an indirect effect of the pathogen which provokes vessel occlusion and cavitation. The knowledge of these genes could constitute an interesting resource for the construction of other tools such as microarrays or exome capture systems by which researchers will be able to analyze the differential expression between tolerant and susceptible genotypes as well as to study the additive effect of DED in a harsher environment caused by climate change. Identified resistance genes or transcription factors from *U. minor* could represent interesting tools for genetic manipulation of elm as a way to combat the disease; for instance, transgenic American elms overexpressing an antimicrobial peptide show reduced DED symptoms (Newhouse et al., 2007). Also a large set of SNPs markers have been discovered in this work. A lot of them are localized in genes putatively involved in response to fungus stimulus and could also be associated with genotypes showing different degrees of tolerance to DED. Thus, SNP analysis could also be designed to look for associations between one or few candidate genes and tolerance to DED in association genetics studies. Another interesting possibility would be exploring the transferability of these molecular resources involved in DED tolerance to other species generally susceptible to the disease such as *U. americana, U. glabra*, or *U. laevis*. Finally, the results of this study will help to better understand genome wide patterns of adaptive variation in Ulmaceae and will help to optimize a correct management of genetic resources of these important noble hardwood species.

## Author Contributions

PP performed SNPs validation, analysis of data and drafted the manuscript. MV prepared vegetal material and performed inoculations. MC conceived the research. LG provided funding. CC conceived and performed the research and drafted the paper. All authors contributed to and approved the final version of the manuscript.

## Conflict of Interest Statement

The authors declare that the research was conducted in the absence of any commercial or financial relationships that could be construed as a potential conflict of interest.
