# Supplementary material for: Massive sequencing of Ulmus minor’s transcriptome provides new molecular tools for a genus under the constant threat of Dutch elm disease
Source: Front Plant Sci. 2015 Jul 20;6:541. doi: 10.3389/fpls.2015.00541 (PMC4507047; doi:10.3389/fpls.2015.00541)
Supplement: Supplementary file 1 [file Table_1.PDF]

Massive sequencing of Ulmus minor's transcriptome provides new molecular tools for a genus under the constant threat of Dutch elm disease

P. Perdiguero [(1,2)], M. Venturas [(1)], M.T Cervera [(2,3)], L. Gil [(1,2)], C. Collada * [(1,2)]

[1] GENFOR Grupo de Investigación en Genética, Fisiología e Historia Forestal, Universidad Politécnica de Madrid. E-28040 Madrid, Spain.
[2] Unidad Mixta de Genómica y Ecofisiología Forestal, INIA/UPM, Madrid, Spain
[3] INIA. Centro de Investigación Forestal. Departamento de Ecología y Genética. Carretera de La Coruña km 7,5 28040 Madrid, Spain

Correspondence:
Carmen Collada, GENFOR Grupo de Investigación en Genética, Fisiología e Historia Forestal
Universidad Politécnica de Madrid, Ciudad Universitaria s/n, E-28040, Madrid, Spain.
carmen.collada@upm.es

Supplementary table 1. Primers pairs used for SNPs validation

Sequence ID	Forward 5´-3´	Reverse 5´-3´
contig02491	CCAAGCCGTGATTAAGGAAA	AACAAATTCAAACTAACAGATACCAAA
contig25532	ACAACTCACGCTACGCCAAA	TGGCTGGAGGAAGCATATCA
isotig02731	AAGCAAGGAGCGGATAGACA	GACCAGGCCCTGTACAAAGT
isotig11158	GAGAAACAAAGCAAACTGAGCA	TGCCAAGAGAAATGACATCG
isotig11256	GGTGTTCCCAAAAAATTGCAG	ATTGCTCACATCAGGCAACC
isotig11583	ATCTCTTGGCCCGTGGTATT	GCACAAAATGAGTTCAAACAACA
isotig13076	GACATCCACGAAACTAAGAGAGC	AAAAACCAATTACCTTGAAATTATGC
isotig13078	GCTCGACACAACTTTGGACA	TCCAAATTTCAATCCAGCCTA
isotig13259	GCGAGGTTCGGGAACAAAT	TCTCTTGCATCACAGCCAAAA
isotig13272	AGATCGAACCCGACAGCATT	GAAAAAGATATCACACCTCACAGCA
isotig18769	GTCCGGCGATACTTGTCTA	GCCCTGGATCTTCTTCAACC
isotig19674	ATCATCGTGAAAGGCACTCC	CACTGAACTAATGTTAGCATCAAGA
isotig20092	CTTCCCAAGCTGGTATAGTTGC	ATCACGCCAAAAATAGTTCACA
isotig20847	GAATTGAAGACCAAGTCAGTGG	CCTTCCCTTTCTTCTGTCCA
